# Correlation analysis of ^11^C-CFT PET/CT parameters with clinical characteristics of patients with early to mid-stage Parkinson’s disease

**DOI:** 10.1186/s12880-025-02081-6

**Published:** 2025-11-28

**Authors:** Tao Zhu, Rui Zuo, Zhu Xia, Wenbo Li, Lili Guan, Jing Chen, Lu Xu, Hua Pang

**Affiliations:** Department of Nuclear Medicine, The First Affiliated Hospital of Chongqing Medical University, Chongqing, China

**Keywords:** Parkinson’s disease, Positron emission tomography, UPDRS score, ^11^C-CFT

## Abstract

**Objective:**

This study aims to explore the correlation between ^11^C-CFT PET/CT imaging parameters and clinical characteristics of PD patients.

**Methods:**

This study retrospectively included 69 PD patients and 10 healthy controls who had undergone ^11^C-CFT imaging to analyze the correlation between ^11^C-CFT imaging parameters and clinical features. The PD patients were divided into TD and PIGD groups based on the TD/PIGD score ratio, and H-Y stage 1–2 and H-Y stage 2.5-3 groups according to the H-Y stage. The differences in ^11^C-CFT parameters were compared between the groups.

**Results:**

After FDR correction for 160 statistical tests, a strong negative correlation was identified between age and both the volume (*r* = -0.471, q < 0.01) and surface area (*r* = -0.474, q < 0.01) of the caudate nucleus. While several correlations between imaging parameters and clinical scores (e.g., UPDRS, bradykinesia) were observed at the nominal significance level (uncorrected *P* < 0.05), none of these associations remained statistically significant after FDR correction.

**Conclusions:**

The most robust finding of this study is a significant age-related decline in caudate nucleus morphology in early to mid-stage PD. After rigorous statistical correction for multiple comparisons, no other significant correlations were found between dopaminergic PET/CT parameters and clinical scores.

## Introduction

Parkinson’s disease (PD) is the second most prevalent neurological disorder, following Alzheimer’s disease [1]. According to the Global Burden of Disease Study 2016, approximately 23% of the worldwide PD population resides in China, and it is projected that by 2030, this proportion will increase to 50% [2, 3]. The primary neuropathological alterations involve the degradation of the nigrostriatal dopaminergic projection pathway and the loss of dopaminergic neurons, resulting in degenerative lesions within the central nervous system. PD predominantly affects the motor function of individuals, resulting in clinical manifestations such as bradykinesia, muscular rigidity, rest tremor, and postural and gait impairment [4]. At present, there is a shortage of dependable and objective measures to evaluate the extent of PD. The Unified Parkinson’s Disease Rating Scale (UPDRS) is frequently employed in clinical settings to evaluate the extent of PD. However, the scoring categories are intricate and convoluted, the process is time-intensive, and the reproducibility is poor [5, 6]. The scoring is influenced by the patient’s medication status and the subjective factors of the scorer.

^11^C-N-2-carbomethoxy-3-(4-fluorophenyl)-tropane (^11^C-CFT) is a dopamine transporter imaging agent that binds specifically to the presynaptic membrane dopamine transporter (DAT). The imaging results can intuitively reflect the functional condition of the dopaminergic system [7, 8]. In the clinical diagnosis of PD, ^11^C-CFT PET/CT imaging provides a new direction for the early diagnosis and treatment of the disease, and differences in the amount of DAT binding in different parts of the striatum in PD patients are a reliable and objective indicator for the early diagnosis of PD. In this research, ^11^C-CFT PET/CT imaging was used to analyze the imaging parameters and clinical data of patients with PD to assess PD progression as an alternative to clinical grading.

## Materials and methods

### Clinical materials

The PET/CT imaging and clinical data of 69 patients with PD who had ^11^C-CFT PET testing and were clinically diagnosed between May 2020 and August 2023 in the Department of Nuclear Medicine of the First Affiliated Hospital of Chongqing Medical University were retrospectively analyzed. The patients were diagnosed by neurologists following the MDS clinical diagnostic criteria for clinically established Parkinson’s disease [9], this process inherently involved the systematic exclusion of patients who presented with any absolute exclusion criteria or unexplained red flags. Furthermore, to ensure a homogeneous cohort and minimize confounding factors, the following additional exclusion criteria were applied: (1)various secondary Parkinson’s syndromes; (2)a history of psychiatric illnesses such as severe depression, anxiety, and schizophrenia; (3) a history of stroke, traumatic brain injury, or other brain diseases.

Subjects’ ages, genders, disease durations, and educational levels were recorded. The clinical staging, motor function, and mental status of all PD patients were assessed using the Modified Hoehn-Yahr (H-Y) staging scale, the Unified Parkinson’s Disease Rating Scale (UPDRS) [10], and the Mini-Mental State Examination (MMSE), respectively. The motor function scores included UPDRS III 3.1–3.9 3.10–3.18; Tremor-Dominant (TD) scores included UPDRS II 2.10, UPDRS III 3.15–3.18; Postural Instability and Gait Difficulty (PIGD) scores included UPDRS II 2.12–2.13, UPDRS III. 3.10–3.12 [11]; rigidity scores included UPDRS III.3.3; and bradykinesia scores included UPDRS II 2.4–2.9, UPDRS III.3.2 3.4–3.8 3.14. Subjects were divided into TD and PIGD groups based on the ratio of their TD/PIGD scores. Those with a ratio ≥ 1.15 were classified as TD, while those with a ratio ≤ 0.90 were classified as PIGD [11]. The remaining subjects were classified as an indeterminate type. The subjects were then classified into the H-Y stage 1–2 early group and the H-Y stage 2.5-3 intermediate group based on the H-Y stage. Ten healthy subjects were selected as the control group.

### ^11^C-CFT PET/CT imaging

^11^C-CFT was prepared by the Department of Nuclear Medicine of our hospital with radiochemical purity > 95%. Before the ^11^C-CFT PET/CT scan, the subject had ceased taking dopa agonists for 72 h and anti-PD medications for 12 h. The head scan was performed using a Philips Gemini TF 64 PET/CT scanner. Before the scan, the participant rested for 30 min in a quiet room and was injected with the molecular probe ^11^C-CFT 555–740 MBq. The head scan was performed after continuing to rest quietly for 40–60 min. The subject was lying supine on the examination bed, with the head fixed on a headrest. The scanning area covered the entire head, and the head was acquired in one bed. The scanning range included the entire skull, at a voltage of 120 keV, current of 100 mA, and slice thickness of 3–5 mm.PET was performed in a three-dimensional model in the same visual field, and the acquisition time was 15–20 min. Subsequently, CT data were utilized for attenuation correction of PET images. Iterative reconstruction was performed using Philips TOF PET software with a reconstruction slice thickness of 4 mm, obtaining cross-sectional, coronal, and sagittal CT, PET, and PET/CT fusion images of the head, respectively.

### ^11^C-CFT PET/CT image analysis

The 3DSlicer software was utilized to employ the region of interest (ROI) method in delineating certain areas, namely the bilateral caudate nucleus, putamen, anterior putamen, and posterior putamen, on PET/CT fusion images. The putamen was subdivided into anterior and posterior compartments on the axial plane using a consistent anatomical landmark: the anterior commissure. For each ROI, the software provided the maximum standardized uptake value (SUVmax) from the PET data, and also calculated the geometric properties (volume and surface area) of the defined three-dimensional region. Cerebellar planes lacking dopamine transporter protein (DAT) distribution were selected as background reference areas. DAT distribution was calculated semiquantitatively according to the formula: ^11^C-CFT uptake value = (SUVmax of each region/SUVmax of the cerebellum)-1 [5]. The SUVmax (maximum standardized uptake value), as well as the volume and surface area of the caudate nucleus, putamen, anterior putamen, and posterior putamen, were calculated by averaging the measurements from both the left and right sides of the corresponding structures.

### Statistical processing

The data was analyzed using SPSS 27.0 software. Normally distributed measurements were expressed as x ± s, while non-normally distributed measures were expressed as M(P25, P75). To compare between groups, t-tests or nonparametric tests were used for independent samples. Pearson’s or Spearman’s correlation was used to analyze the correlation between striatal ^11^C-CFT uptake and clinical data of PD patients. All enrolled PD patients (including those classified as having an indeterminate motor subtype) were included in the comprehensive correlation analysis. *P* < 0.05 is considered statistically significant. To account for the multiple comparisons inherent in the correlation analysis between multiple imaging parameters and clinical scores, the False Discovery Rate (FDR) correction was applied using the Benjamini-Hochberg procedure. The FDR-adjusted *q*-value of < 0.05 was set as the threshold for statistical significance for all correlation tests, in addition to the uncorrected P-value.

## Results

### General clinical information

Table 1 shows the demographic and clinical information for all individuals. This study included 69 individuals with PD and 10 healthy volunteers. The PD group included 34 males and 35 females, with an average age of 61.99 ± 9.46 years. The control group included 4 males and 6 females, with an average age of 62.40 ± 10.53 years. There was no significant difference in gender or age distribution between the PD and control groups. There was a positive correlation between the MMSE scores and the educational level in the PD group (*r* = 0.507, *P* < 0.001), H-Y stage was positively correlated with the UPDRS score (*r* = 0.699, *P* < 0.001), UPDRS-III score (*r* = 0.732, *P* < 0.001), PIGD score (*r* = 0.600, *P* < 0.001), rigidity score (*r* = 0.566, *P* < 0.001), and bradykinesia score (*r* = 0.729, *P* < 0.001).

**Table 1:** Demographic and clinical information of the PD and control groups

	Control group (*n* = 10)	PD group (*n* = 69)	*P*
Sex (m/f, n/n)	4/6	34/35	0.583
Age (x ± s, years)	62.4 ± 10.53	61.99 ± 9.46	0.899
Disease Duration [M (P25, P75), months]	-	24.00 (12.00,48.00)	-
Below elementary school	-	2	-
Elementary school	-	7	-
Junior high school	-	22	-
Senior high school	-	19	-
College	-	18	-
Family history of PD (n)	-	7	-
Hypertension (n)	-	6	-
Diabetes (n)	-	2	-
H-Y stage [(1–2) /(2.5-3), n/n]	-	57/12	-
TD group/PIGD group	-	30/36	-

### Correlation analysis between ^11^C-CFT PET/CT parameters and clinical features

Table 2 summarizes the correlations between ^11^C-CFT parameters and clinical features within the PD patient cohort. After FDR correction for multiple comparisons, a negative correlation was observed between age and the volume of the caudate nucleus (*r* = -0.471, *q* < 0.01), and between age and the surface area of the caudate nucleus (*r* = -0.474, *q* < 0.01). The correlations for all other ^11^C-CFT parameters with clinical scores were not significant after FDR correction (*q* > 0.05).

**Table 2:** Correlation coefficients for correlation analysis of ^11^C-CFT PET/CT parameters with clinical characteristics

	Age	*q*	Disease duration	*q*	UPDRS score	q	UPDRS-III score	q	TD score	q	PIGD score	q	Rigidity score	q	Bradykinesia	q	H-Y stage	q	MMSE score	q
SUVmax of the caudate nucleus	0.003	0.992	-0.047	0.803	-0.204	0.271	-0.163	0.369	-0.088	0.625	0.036	0.860	-0.138	0.455	-0.234	0.207	-0.084	0.625	-0.002	0.993
SUVmax of the putamen	0.027	0.904	-0.087	0.625	-0.226	0.225	-0.177	0.327	-0.085	0.625	0.001	0.993	-0.148	0.416	-0.244	0.207	-0.086	0.625	-0.018	0.947
SUVmax of the anterior putamen	0.029	0.900	-0.084	0.639	-0.222	0.227	-0.171	0.348	-0.081	0.639	0.005	0.989	-0.145	0.427	-0.239	0.207	-0.088	0.625	-0.020	0.944
SUVmax of the posterior putamen	0.065	0.712	-0.125	0.505	-0.256	0.207	-0.211	0.254	-0.121	0.505	-0.017	0.947	-0.155	0.391	-0.267	0.193	-0.107	0.556	-0.035	0.862
^11^C-CFT uptake in the caudate nucleus	-0.179	0.325	-0.008	0.988	-0.188	0.310	-0.147	0.416	0.197	0.280	-0.072	0.678	-0.105	0.564	-0.234	0.207	-0.153	0.397	0.185	0.320
^11^C-CFT uptake in the putamen	-0.045	0.803	-0.133	0.488	-0.192	0.299	-0.097	0.592	0.218	0.235	-0.138	0.455	-0.057	0.748	-0.178	0.325	-0.087	0.625	0.111	0.551
^11^C-CFT uptake in the anterior putamen	-0.048	0.792	-0.133	0.488	-0.182	0.320	-0.085	0.625	0.223	0.227	-0.128	0.488	-0.050	0.784	-0.168	0.354	-0.092	0.621	0.109	0.556
^11^C-CFT uptake in the posterior putamen	0.057	0.748	-0.279	0.193	-0.239	0.207	-0.159	0.383	0.075	0.663	-0.124	0.494	-0.088	0.625	-0.202	0.271	-0.116	0.519	0.080	0.644
Surface area of the caudate nucleus	-0.474	0.000*	0.103	0.592	-0.289	0.171	-0.184	0.320	0.264	0.193	-0.207	0.271	-0.116	0.519	-0.293	0.171	-0.181	0.322	0.332	0.107
Surface area of the putamen	-0.010	0.986	-0.212	0.271	-0.329	0.107	-0.200	0.278	0.127	0.488	-0.199	0.279	-0.101	0.207	-0.316	0.107	-0.263	0.193	0.062	0.730
Surface area of the anterior putamen	-0.170	0.350	-0.105	0.581	-0.318	0.107	-0.236	0.207	0.158	0.383	-0.168	0.354	-0.166	0.357	-0.363	0.080	-0.265	0.193	0.252	0.207
Surface area of the posterior putamen	0.221	0.227	-0.269	0.198	-0.189	0.310	-0.071	0.679	0.015	0.959	-0.119	0.509	0.057	0.748	-0.113	0.532	-0.127	0.488	-0.098	0.592
Volume of the caudate nucleus	-0.471	0.000*	0.101	0.592	-0.292	0.171	-0.187	0.310	0.253	0.207	-0.231	0.212	-0.141	0.448	-0.279	0.188	-0.156	0.391	0.319	0.107
Volume of the putamen	-0.006	0.989	-0.214	0.278	-0.349	0.080	-0.248	0.207	0.126	0.488	-0.243	0.207	-0.174	0.340	-0.340	0.091	-0.273	0.193	0.025	0.913
Volume of the anterior putamen	-0.235	0.207	-0.071	0.694	-0.352	0.080	-0.270	0.193	0.198	0.279	-0.230	0.212	-0.214	0.250	-0.398	0.053	-0.284	0.180	0.246	0.207
Volume of the posterior putamen	0.247	0.207	-0.262	0.207	-0.221	0.227	-0.123	0.494	-0.008	0.988	-0.127	0.488	-0.005	0.989	-0.163	0.369	-0.153	0.397	-0.128	0.488

TD = Tremor-Dominant, PIGD = Postural Instability and Gait Difficulty; * at the 0.05 level, the correlation is significant

### Comparison between groups

There were significant differences in ^11^C-CFT parameters (except the surface area and volume of the caudate nucleus and anterior putamen) between TD, H-Y stage 1–2, H-Y stage 2.5-3, and control groups. There were significant differences in ^11^C-CFT parameters (except SUVmax of the caudate nucleus, the surface area and volume of the caudate nucleus, and anterior putamen) between PIGD and control groups.

**Fig. 1:**
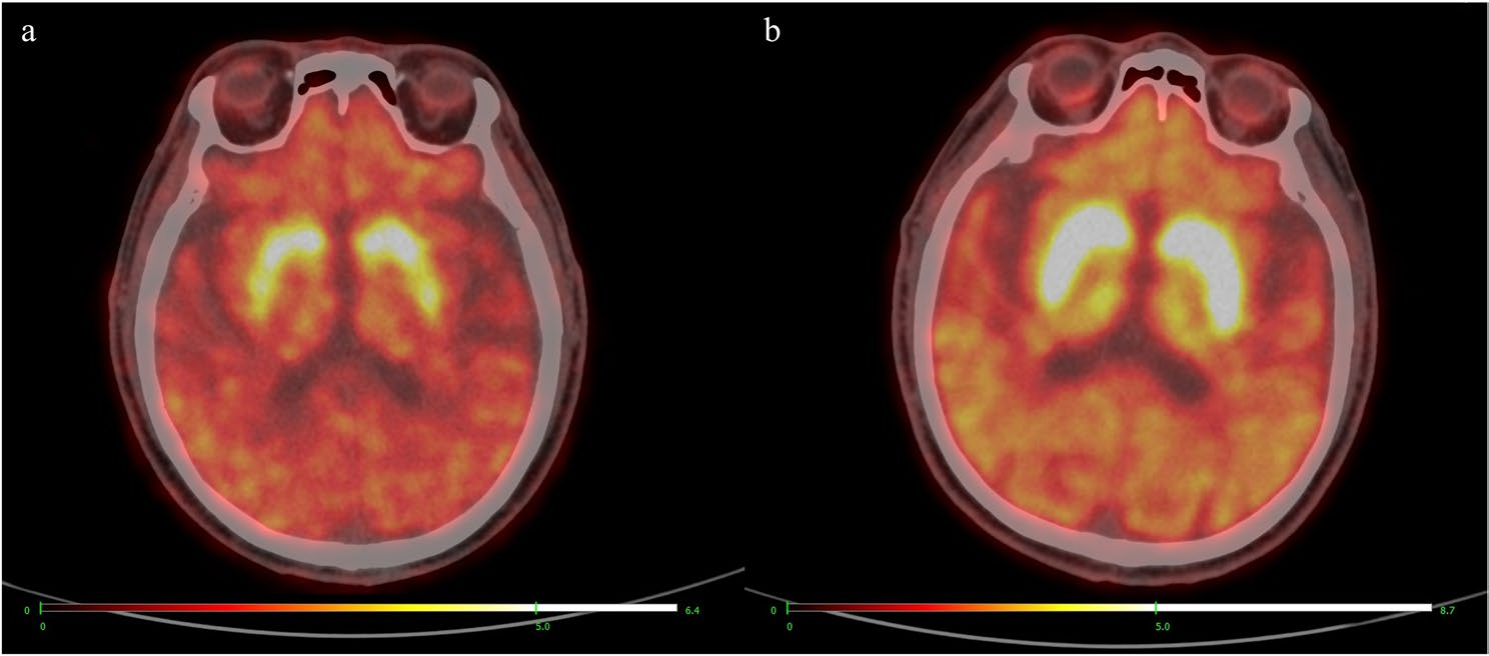
Typical striatum ^11^C-CFT PET/CT imaging. **a**. PET/CT fusion images of PD patients; **b**. PET/CT fusion images of healthy controls. Images are displayed on an identical fixed SUVmax scale for accurate visual comparison

**Fig. 2:**
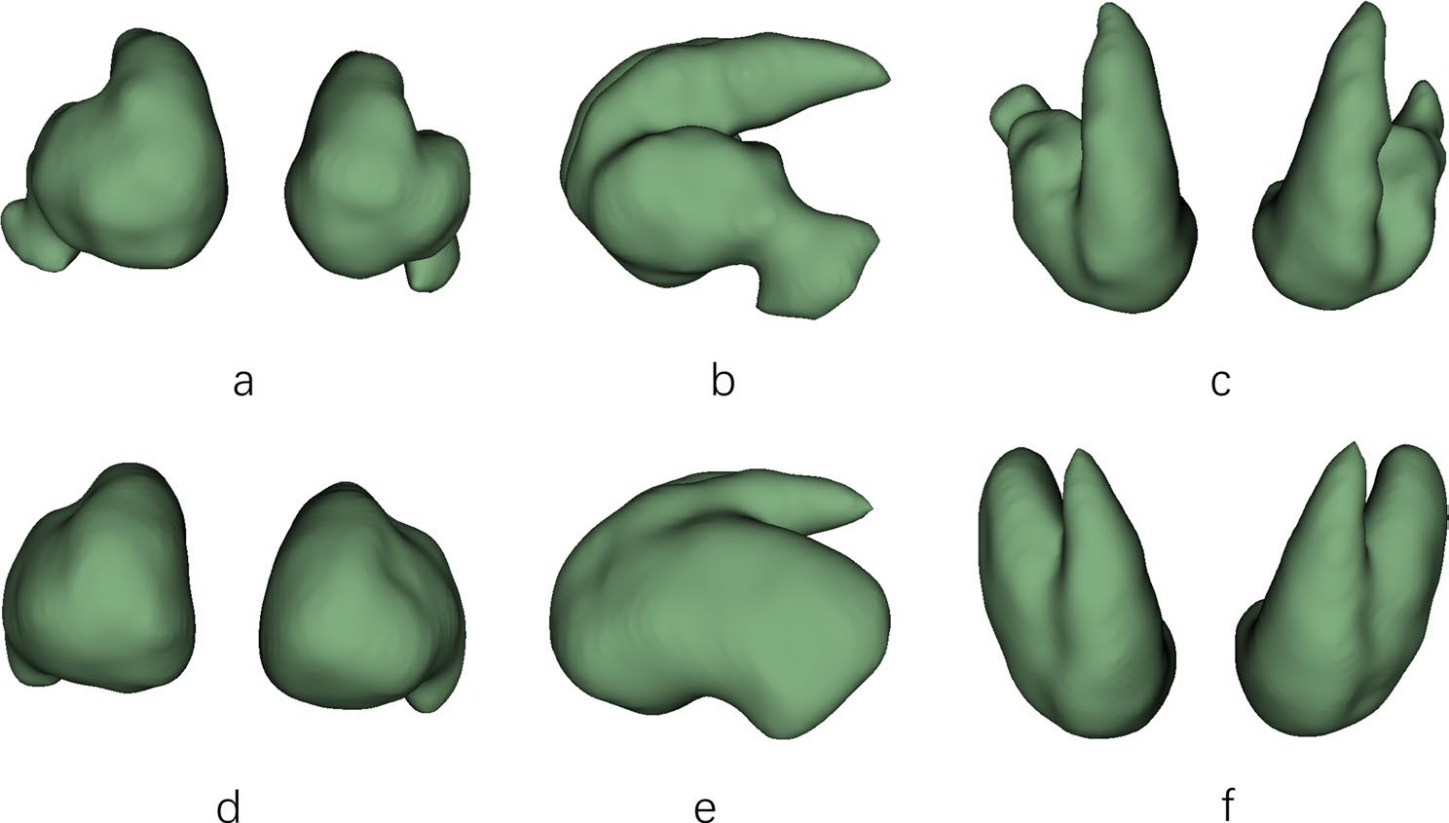
Typical striatum ^11^C-CFT PET/CT 3D graphics. **a**-**c**. Frontal, lateral, and top views of the striatum of PD patients, respectively; **d**-**f**. Frontal, lateral, and top views of the healthy control striatum, respectively

The ^11^C-CFT uptake values of the putamen and anterior putamen in the TD group were higher than those in the PIGD group, and the rest of the ^11^C-CFT parameters were not significantly different (Table 3). There were no significant differences in the ^11^C-CFT parameters between the H-Y stage 1–2 group and the H-Y stage 2.5-3 group (Table 4). Figure 1 shows typical ^11^C-CFT PET/CT images of the striatum in the PD and control groups, whereas Fig. 2 shows 3D graphics of the striatum’s ^11^C-CFT PET/CT uptake.

**Table 3:** Comparison of ^11^C-CFT PET/CT parameters in TD, PIGD, and control groups

	TD group (*n*=30)	PIGD group (*n*=36)	Control group (*n*=10)	*P*
SUVmax of the caudate nucleus	10.44±7.52	10.90±6.67	13.86±4.66	<0.05^a)^
SUVmax of the putamen	9.93±7.57	9.77±6.02	14.83±5.09	<0.05^b)^
SUVmax of the anterior putamen	9.90±7.58	9.73±5.99	14.78±5.14	<0.05^b)^
SUVmax of the poterior putamen	7.56±6.74	7.53±4.84	14.27±4.68	<0.05^b)^
^11^C-CFT uptake in the caudate nucleus	3.04±0.77	2.69±0.92	3.84±0.40	<0.05^b)^
^11^C-CFT uptake in the putamen	2.77±0.79	2.34±0.89	4.18±0.55	<0.05^c)^
^11^C-CFT uptake in the anterior putamen	2.75±0.79	2.33±0.88	4.16±0.57	<0.05^c)^
^11^C-CFT uptake in the posterior putamen	1.81±0.68	1.58±0.81	4.00±0.47	<0.05^b)^
Surface area of the caudate nucleus	2021.68±331.81	1976.45±314.68	2033.53±96.04	-
Surface area of the putamen	2063.65±286.90	2053.16±329.89	2308.84±75.75	<0.05^b)^
Surface area of the anterior putamen	1425.09±241.75	1382.10±215.87	1378.80±126.40	-
Surface area of the poterior putamen	985.78±229.93	1020.16±304.28	1402.72±78.56	<0.05^b)^
Volume of the caudate nucleus	5776.01±1277.13	5445.21±1231.57	5928.64±418.10	-
Volume of the putamen	5973.45±1377.89	5787.28±1477.95	7646.93±395.36	<0.05^b)^
Volume of the anterior putamen	3932.96±952.68	3646.36±847.81	3720.13±564.27	-
Volume of the posterior putamen	2073.24±701.47	2209.21±889.26	3926.80±335.91	<0.05^b)^

(a) *P* < 0.05 for TD group vs. Control group; (b) *P* < 0.05 for both TD and PIGD groups vs. Control group; (c) *P* < 0.05 for all pairwise comparisons between TD, PIGD, and Control groups; d) All data are presented as the mean ± standard deviation

**Table 4:** Comparison of ^11^C-CFT PET/CT parameters in H-Y stage 1-2, H-Y stage 2.5-3 and control group

	H-Y stage 1-2 (*n*=57)	H-Y stage 2.5-3 (*n*=12)	Control group (*n*=10)	*P*
SUVmax of the caudate nucleus	10.76±7.34	9.43±4.38	13.86±4.66	<0.05^a)^
SUVmax of the putamen	9.90±7.00	8.64±4.47	14.83±5.09	<0.05^a)^
SUVmax of the anterior putamen	9.87±6.98	8.60±4.50	14.78±5.14	<0.05^a)^
SUVmax of the poterior putamen	7.69±6.04	6.02±3.03	14.27±4.68	<0.05^a)^
^11^C-CFT uptake in the caudate nucleus	2.88±0.80	2.72±1.08	3.84±0.40	<0.05^a)^
^11^C-CFT uptake in the putamen	2.56±0.82	2.36±1.01	4.18±0.55	<0.05^a)^
^11^C-CFT uptake in the anterior putamen	2.55±0.81	2.35±1.03	4.16±0.57	<0.05^a)^
^11^C-CFT uptake in the posterior putamen	1.74±0.75	1.33±0.61	4.00±0.47	<0.05^a)^
Surface area of the caudate nucleus	1993.16±311.4	2019.28±340.09	2033.53±96.04	-
Surface area of the putamen	2079.68±301.67	1894.69±297.05	2308.84±75.75	<0.05^a)^
Surface area of the anterior putamen	1426.93±229.05	1285.81±150.58	1378.80±126.40	-
Surface area of the poterior putamen	1017.13±270.87	884.02±277.60	1402.72±78.56	<0.05^a)^
Volume of the caudate nucleus	5584.36±1207.91	5660.62±1369.62	5928.64±418.10	-
Volume of the putamen	5974.68±1418.78	5126.46±1204.16	7646.93±395.36	<0.05^a)^
Volume of the anterior putamen	3859.72±898.29	3369.04±700.06	3720.13±564.27	-
Volume of the posterior putamen	2195.46±816.70	1757.42±704.98	3926.80±335.91	<0.05^a)^

(a) *P* < 0.05, for both H-Y stage 1-2 group and H-Y stage 2.5-3 group vs. Control group; (b) All data are presented as the mean ± standard deviation

## Discussion

PD is caused primarily by the malfunctioning of dopaminergic neurons in the brain, which have their primary projections in the substantia nigra-striatal pathway, a crucial component of the brain for regulating movement and coordinating actions. Dopamine in the brain is primarily concentrated in bilateral striatal regions [1]. Gradual loss of dopaminergic neurons in the substantia nigra reduces striatal dopamine levels, affecting motor function and causing a variety of movement disorders. ^11^C-CFT uptake is mainly distributed in the caudate nucleus and putamen regions of the brain, higher than other brain regions.

In this investigation, our primary and most statistically robust finding within the PD patient cohort was the strong negative correlation between patient age and the morphology of the caudate nucleus (volume: *r* = -0.471, q < 0.01; surface area: *r* = -0.474, q < 0.01). This age-related structural decline is consistent with established patterns of brain aging and has been observed in the context of neurodegenerative processes in PD [12, 13]. While our analysis cannot establish whether this association is specific to PD without a direct case-control correlation comparison, this result underscores that age-associated morphological changes in the striatum remain a prominent and measurable feature in early to mid-stage PD.

Prior to multiple comparisons correction, our analysis, in line with existing literature [3, 14], suggested several correlations between dopaminergic parameters and clinical scores. For instance, negative correlations were observed between the SUVmax of the posterior putamen and both UPDRS and bradykinesia scores, and between ^11^C-CFT uptake in the posterior putamen and disease duration. These uncorrected findings aligned with the established pathological model of PD, wherein the mid-posterior putamen is often the earliest and most severely affected region, receiving dense projections from the ventral substantia nigra and exhibiting a faster decline in DAT availability [14].

However, it is crucial to emphasize that none of these correlations, nor those between other ^11^C-CFT parameters and a wide range of clinical scores (e.g., UPDRS, bradykinesia, H-Y stage), remained statistically significant after FDR correction. The lack of significant differences in the surface area and volume of the caudate nucleus and anterior putamen between PD patients and healthy controls in our study can be more confidently attributed to the early disease stage of our cohort, where significant atrophy in these regions may not yet have manifested [15, 16], rather than to specific clinical correlations.

Similarly, the initial, uncorrected positive correlations between the volume and surface area of the caudate nucleus and MMSE scores, and between the anterior putamen and MMSE scores, suggested a link between striatal morphology and cognitive function [17–19]. While biologically plausible, these relationships did not survive multiple comparisons correction and therefore cannot be considered statistically robust findings in our cohort. This calls for caution in interpreting such cross-sectional correlations without appropriate statistical safeguards.

Consistent with some previous studies [20–22], ^11^C-CFT uptake parameters in our data were not robustly correlated with tremor dominance (TD) after correction. The initially observed positive correlation between TD scores and caudate nucleus morphology was not statistically robust. This finding is mechanistically supported by evidence suggesting that Parkinsonian tremor may originate from alterations in the cortex-STN-GPi-thalamus-cortex loop, a network that largely excludes the striatum [23]. This provides a pathophysiological explanation for the lack of a strong, direct relationship between striatal integrity, as measured by ^11^C-CFT PET/CT and structural MRI, and tremor severity.

Regarding motor subtypes, the observations that the TD group had higher ^11^C-CFT uptake values in the putamen than the PIGD group, and the negative association between PIGD score and putamen volume, were consistent with the recognized more rapid disease progression and greater non-dopaminergic pathology in the PIGD subtype [24–27]. However, the failure of this negative association to withstand multiple comparison correction suggests that the discriminative power of these specific imaging metrics for subtyping in early to mid-stage PD may be limited in a cross-sectional design.

No significant correlation was found between the volume of the posterior putamen and age after FDR correction. Although a positive trend was observed in the uncorrected analysis—contrasting with the strong negative correlation in the caudate nucleus—this finding did not survive rigorous statistical control. This could be related to complex regional variations in the interaction between age and PD pathology. For instance, disproportionate putamen alterations might be more obvious in patients with younger onset of PD [28], and studies have shown differential relationships between age and DAT binding in the caudate versus the putamen [29]. This merits further investigation in cohorts specifically stratified by age of onset.

In this work, the relationship between multiple ^11^C-CFT PET/CT parameters and clinical data was thoroughly investigated with rigorous statistical control. The most definitive finding was the significant correlation between age and caudate nucleus morphology. The study introduces the volume and surface area of individual striatal regions as additional metrics, and the concordance between these geometrically correlated parameters reinforces the robustness of the morphological data. However, the overarching conclusion is that initial, uncorrected correlations between imaging parameters and clinical scores were not robust to multiple comparisons correction. This underscores the capability of ^11^C-CFT PET/CT to provide complementary quantitative metrics but also highlights the critical need for stringent statistical practices in exploratory biomarker research. It expands the possibilities for clinical PD evaluation by setting a higher standard of evidence. A limitation of our study is the use of bilaterally averaged ROI values. This approach was chosen as our primary aim was to explore the overall correlation between striatal parameters and clinical characteristics, rather than to investigate lateralized symptoms. Consequently, our analysis does not capture the known hemispheric asymmetry of dopaminergic loss in PD. A further limitation is the statistical power of our study. With a sample size of 69 patients, our analysis was likely underpowered to detect modest correlation effects (|r| < 0.35) after applying the stringent FDR correction for 160 statistical tests. Future studies with larger sample sizes and longitudinal designs, as well as those incorporating asymmetry indices or voxel-based analyses, are essential to validate more associations, account for clinical heterogeneity, and determine the utility of these multi-parametric biomarkers in predicting PD progression.

## Conclusion

The most robust finding of this study is the association between age and caudate nucleus structure in PD. This study did not establish significant correlations between the ^11^C-CFT parameters and clinical scores after multiple comparisons correction. This highlights the complexity of linking imaging biomarkers to symptoms and calls for future validation in larger cohorts.

## Data Availability

Data and material in the study are available from the corresponding author on reasonable request.

## Abbreviations

^11^C-CFT: ^11^C-N-2-carbomethoxy-3-(4-fluorophenyl)-tropane
PET/CT: Positron Emission Tomography/Computed Tomography
PD: Parkinson’s disease
TD: Tremor-Dominant
PIGD: Postural Instability and Gait Difficulty
H-Y: Hoehn-Yahr
FDR: False Discovery Rate
UPDRS: Unified Parkinson’s Disease Rating Scale
DAT: Dopamine transporter
MDS: Movement Disorder Society
MMSE: Mini-Mental State Examination
ROI: Region of interest
SUVmax: Maximum Standardized Uptake Value
